# Statistical Redundancy Testing for Improved Gene Selection in Cancer Classification Using Microarray Data

**Published:** 2007-02-06

**Authors:** Simin Hu, J. Sunil Rao

**Affiliations:** Department of Epidemiology and Biostatistics, Case Western Reserve University, Cleveland, Ohio, 44106

**Keywords:** gene selection, microarray, cancer classification, statistical redundancy

## Abstract

In gene selection for cancer classification using microarray data, we define an eigenvalue-ratio statistic to measure a gene’s contribution to the joint discriminability when this gene is included into a set of genes. Based on this eigenvalue-ratio statistic, we define a novel hypothesis testing for gene statistical redundancy and propose two gene selection methods. Simulation studies illustrate the agreement between statistical redundancy testing and gene selection methods. Real data examples show the proposed gene selection methods can select a compact gene subset which can not only be used to build high quality cancer classifiers but also show biological relevance.

## Introduction

Gene expression profiling has been successfully used to identify potential cancer diagnostic and therapeutic targets in the past few years. Since there are often a large number of potential genes, an important issue is to find a small subset of genes that are differentially expressed between different clinical outcomes or related to cancer patients survival time, and thus can be used to build prognosis predictors. While gene selection for cancer classification has been studied extensively in the literature, a less studied but very important concept is the redundancy problem. Commonly used gene selection methods include univariate methods and multivariate methods. Since genes with extremely differential expression levels are believed to have differential behaviors between different cancer types or subtypes, univariate ranking methods are based on genes’ individual ability to separate between two or more classes of tissues. These methods rank individual genes according to a criterion such as *t*-statistic or Wilcoxon rank sum test statistic for two-class classification, and F-statistic or Kruskal-Wallis test statistic for multiple-class classification, then select top ranked genes for classification. These methods do not take the correlation between genes into consideration and introduce a great deal of redundancy because strongly correlated genes with similar expression levels are very likely to be selected together. When a selected gene subset contains redundant information, it can not be maximally representative of the targeted cancer types like a subset of the same size but without redundancy, or the efficiency and the performance of a classifier based on this set can be improved by removing those redundant genes. Multivariate methods select genes by evaluating the joint discriminability of a set of genes, i.e. the predictive accuracy with a predetermined classification Algorithm. One example of multivariate methods is subset selection method which finds an optimal subset with minimum classification error by searching for *P*!/[*R*!(*P* – *R*)!] candidate subsets of size *R* given a set of *P* genes. Another example is Recursive Feature Elimination (RFE) which recursively removes genes with smallest ranking criterion, evaluates the resulted nested subsets and optimizes the subset for a given classifier. Such kinds of multivariate methods can remove both irrelevant and redundant genes when searching for an optimal minimum subset of genes for a good classifier. However, these methods need to build a classifier for each searched subset which might be very computationally intensive especially for high-dimensional data. Zou and Hastie (2004) proposed a very efficient regularization method “elastic net” and applied their method to gene selection for microarray classification. Their method does not however take care of redundancy. On the contrary, it actually encourages a grouping effect thus tends to select strongly correlated genes into the model together.

There have been a few gene selection methods based on information theory and developed to explicitly and directly reduce redundancy (e.g. Xing et al. 2001; Yu and Liu, 2004). These methods make use of a Markov blanket filtering procedure to remove redundant genes. Markov blanket filtering is a backward elimination procedure due to Koller and Sahami (1996) and was proved to guarantee a variable removed in an earlier phase will still find a Markov blanket in any later phase. When the information that a gene has about the targeted groups can be subsumed by some small subset of the other genes called a Markov blanket, this gene can be removed from the subset without compromising the accuracy of class prediction. Actually, Markov blanket criterion can remove both redundant and irrelevant genes. In Ding and Peng (2003), the selected genes are those having the maximal mutual information with the targeted groups and meeting the minimum redundancy criteria, i.e. those genes’ mutual Euclidean distances are maximized or their pair-wise correlations are minimized. Jaeger et al. (2003) used five kinds of test statistics to rank genes by groups that are retrieved by clustering or correlation and avoided redundancy by selecting one or more highly-ranking genes from each cluster.

To distinguish from the concepts “gene redundancy” in genetic analysis, and “redundancy” and “redundancy analysis” in multivariate statistics, we specify in this paper the redundancy problem to be addressed as “gene statistical redundancy” and the corresponding hypothesis testing as “gene statistical redundancy testing.” For cancer classification, we define an eigenvalue-ratio statistic to measure each gene’s contribution to the joint discriminability when considered within a set of genes. We assume linear relationships between gene expression levels and the targeted classes and apply Fisher linear discriminant analysis method (FLDA) to reduce the data. Since the joint discriminability of a set of genes can be measured by the largest eigenvalues when applying the linear combinations found by maximizing the ratio of between variance and within variance, one gene’s contribution to the joint discriminability of a set of genes can be measured by the change of the eigenvalues when added to this set. In other words, a very small change indicates that this gene has a very small contribution to the set. For each gene, we define the contribution measure as the ratio of the eigenvalue computed after adding this gene to the eigenvalue computed before adding this gene. In the extreme case when a set includes duplicated genes, we get exactly the same eigenvalue whether adding a duplicated gene or not thus the eigenvalue-ratio for a duplicated gene is 1. When a gene’s eigenvalue-ratio is not significantly greater than 1, we conclude that this gene’s contribution to the whole set is not important and is negligible when the other genes are already included in this set. Actually, there may be two reasons for small contribution of a gene to the joint discriminability: 1) this gene has high individual discriminability but the information that this gene has about the targeted classes can be replaced by other genes that have even higher individual discriminability than and strongly correlated gene expression levels with this gene, and 2) this gene has low individual discriminability. Thus a very small change of the eigenvalue indicates either this gene is statistically redundant or statistically less important. For a set of strongly correlated genes with high discriminability, we evaluate such statistical redundancy by bootstrap testing the null hypothesis that eigenvalue-ratio is not significantly greater than 1 versus the alternative. For *K*-class classification, there are at most *K* – 1 non-zero eigenvalues and *K* – 1 corresponding orthogonal discriminant functions. We focus on 2-class classification problems in this paper thus only one meaningful eigenvalue is obtained from each eigenvalue-decomposition of FLDA and only one eigenvalue-ratio needs to be computed for statistical redundancy testing.

In case of perfect multi-collinearity, FLDA will not have a unique discriminant solution. In case of low multi-collinearity, the standardized discriminant function coefficients will not reliably assess the relative importance of the predictor variables. However, the multi-collinearity problem will not be a concern here although strong correlations exist among genes with similar expression levels. The reason is that we do not directly use the discriminant functions but rather the eigenvalue ratios to assess the contribution of each gene.

Based on the eigenvalue-ratio statistic, our first gene selection method “Algorithm 1” filters statistically redundant or statistically less important genes with lower eigenvalue-ratios when *P* ≤ *N* − *K* − 2 ( *P* is the number of genes, *N* is the sample size and *K* is the number of classes). Since the within-class variance matrix in FLDA is singular when *P* ≥ *N* – *K*, we propose a second gene selection method “Algorithm 2” in such cases by introducing DIANA (Divisive Analysis) clustering Algorithm (Kaufman and Rousseeuw, 1990) with (1-Pearson correlation) as the distance measure. Datta and Datta (2003) evaluated and compared the performances of DIANA with five other clustering Algorithms such as hierarchical clustering with correlation, *K*-means, Fanny and Hierarchical clustering with partial least squares and model-based clustering. They found out that both *K*-means and DIANA are most effective in achieving good separation and almost distinct class boundaries, but only the latter shows consistent successful performance under all three validation measures used in their work. An attractive feature of the two algorithms is that it not only explicitly removes statistically redundant genes but also may shed a light on the biological interaction between the selected genes and removed ones. This will be demonstrated with simulation and real data studies. Gene selection with both algorithms is simply gene-filtering according to eigenvalue ratios and the whole filtering process shows to be computationally very efficient. Mean while, we remove those statistically redundant and statistically less important genes and thus we can expect to obtain a compact subset which is more representative than a set with the same size but including statistically redundant genes. We evaluate the joint discriminability of the selected subset by building logistic regression models. For comparison with other gene selection methods that eliminate statistical redundancy, we implement Yu and Liu’s (2004) Fast Correlation-Based Filter (FCBF) algorithm in R and use this algorithm for both simulated and real data examples (Golub et al. 1999; Alon et al. 1999). We also compare our results with Guyon et al.’s (2002) results obtained with Support Vector Machine methods based on Recursive Feature Elimination (SVM RFE). Both simulation and real data studies show that our method is very effective in removing statistical redundancy and select a highly predictive set of genes.

The rest of this paper is organized as follows: in Section 2, we first define the eigenvalue-ratio statistic and describe the bootstrap re-sampling statistical redundancy testing method, and, then present our gene selection methods using the eigenvalue-ratio statistic; Section 3 illustrates results on some simulations; Section 4 gives gene selection results for two benchmark cancer gene expression datasets; and Section 5 contains conclusions and discussion for the future work. Some information for Divisive Analysis (DIANA) clustering Algorithm and FLDA, and, the selected genes for the two real data examples can be found in the Supplementary materials.

## Statistical Redundancy Testing and Gene Selection Algorithms

Let gene expression data set on *P* genes for *N* samples be summarized by an *P* × *N* matrix *X* = (*x_ij_*) where *x_ij_* denotes the expression level of gene *i* in sample *j*. Each sample *j* is thought to originate from a class *k* ∈ {1, …, *K*} and the number of possible classes *K* is known to be fixed.

### Test statistic: eigenvalue-ratio

For gene *i* in a set of genes, let $\lambda_{1}=\frac{\alpha_{1}^{T}S_{B}\alpha_{1}}{\alpha_{1}^{T}S_{w}\alpha_{1}}$ and $\lambda_{1}^{\prime }=\frac{\alpha_{1}^{\prime T}S_{B}^{\prime }\alpha^{\prime }}{\alpha_{1}^{\prime T}S_{w}^{\prime }\alpha^{\prime }}$ be respectively the first largest eigenvalues from eigenvalue-decomposition of FLDA when including this gene in the set or not. *S_B_* and *S′_B_* are between-class variance matrices, *S_W_* and *S′_W_* are within-class covariance matrices, and, the first linear combinations α_1_ and α*′*_1_ are given by the largest eigenvalues λ_1_ and λ*′*_1_ when solving a generalized eigenvalue problem.

To test the statistical redundancy of this gene *i* in separating the cancer classes, we do hypothesis testing *H*_0_: λ_1_ = λ*′*_1_ vs. *H*_1_: λ_1_ = λ*′*_1_ based on a test statistic eigenvalue-ratio λ_1_/λ*′*_1_. So the null hypothesis is that there is no significant difference in discriminability of this set of genes whether including this gene or not. And the alternative hypothesis is that including this gene does improve the discriminability of this set.

When including gene *i* in a set of genes, we denote the *F*-test for fixed effect of one-way analysis of variance on the discriminant scores obtained by applying the first linear combination α_1_ to the data as

$$F_{1}\;\;=\frac{\alpha_{1}^{T}S_{B}\alpha_{1}/(K-1)}{\alpha_{1}^{T}S_{w}\alpha_{1}/(N-K)}$$

which has a non-central F-distribution with degrees of freedom (*K –* 1, *N – K*).

Similarly, we denote the *F*-test when not including gene *i* in the set as

$$F_{1}^{\prime }\;\;=\frac{\alpha_{1}^{\prime T}S_{B}^{\prime }\alpha_{1}^{\prime }/(K-1)}{\alpha_{1}^{\prime T}S_{w}^{\prime }\alpha_{1}^{\prime }/(N-K)}$$

which has a same non-central F-distribution with degrees of freedom (*K* − 1, *N − K*).

So an equivalent test statistic for statistical redundancy testing can be *F*_1_/*F′*_1_ i.e. the ratio of two non-central F distributions, since the F-tests can be written as

$$F_{1}=\lambda_{1}\frac{N-K}{K-1}\;\;\text{and}\;\;F_{1}^{\prime }=\lambda_{1}^{\prime }\frac{N-K}{K-1}$$

Schumann and Bradley (1957) obtained the distribution of the ratio of two independent non-central variance ratios and proposed to use such a ratio as test statistic for comparison of the sensitivities of two independent experimental techniques when assuming a fixed effects analysis of variance. In one-way classifications, let $\sigma_{A}^{2}$ and $\sigma_{e}^{2}$ respectively denote the components of variance between and within groups, sensitivity is measured by $\sigma_{A}^{2}/\sigma_{e}^{2}$. Dar (1962) obtained a large sample test for sensitivities in two identical or similar independent experiments by using a normalizing transformation. Let *MS_A_* and *MS_e_* be the corresponding mean squares. To test the null hypothesis *H*_0_: $\sigma_{A_{1}}^{2}/\sigma_{e_{1}}^{2}=\sigma_{A_{2}}^{2}/\sigma_{e_{2}}^{2}$ for two sensitivities, the test statistic can be written as (*MS*_*A*_1__/*MS*_*e*_1__) × (*MS*_*e*_2__/*MS*_*A*_2__) because both *MS*_*A*_1__/*MS_e_*__1__ and *MS*_*A*_2__/*MS*_*e*_2__ are distributed as (1 + *J* $\sigma_{A}^{2}/\sigma_{e}^{2}$) times a non-central *F* distribution with degrees of freedom (*v_A_*, *v_e_*) for two identical independent experiments with equal group size *J*. Dar (1962) used an equivalent statistic *z*_1_ − *z*_2_ and tested $(z_{1}-z_{2})/\sqrt{1/(v_{A}-1)+1/(v_{e}-1)}$ as a standard normal variable where

$$Z_{i}=\frac{1}{2}\text{log}\;\;(MS_{A_{1}}/MS_{e_{1}})\;(\text{i}=1,2)$$

This test can be applied for two similar experiments as long as the sum of squares of group sizes is constant. Thus it seems very appealing to apply a similar large sample test as that in Dar (1962) for statistical redundancy testing,

$$\begin{array}{l} \frac{1}{2}\text{log}\left(\frac{\lambda_{1}}{\lambda_{1}^{\prime }}\right)=\frac{1}{2}\text{log}\left(\frac{\alpha_{1}^{T}M_{B}\alpha_{1}}{\alpha_{1}^{T}M_{W}\alpha_{1}}\right)\\ \;-\;\frac{1}{2}\text{log}\left(\frac{\alpha_{1}^{\prime T}M_{B}^{\prime }\alpha_{1}^{\prime }}{\alpha_{1}^{\prime T}M_{W}^{\prime }\alpha_{1}^{\prime }}\right) \end{array}$$

where $\alpha_{1}^{T}M_{B}\alpha_{1}$ and $\alpha_{1}^{T}M_{W}\alpha_{1}$, $\alpha_{1}^{\prime T}M_{B}^{\prime }\alpha_{1}^{\prime }$ and $\alpha_{1}^{\prime T}M_{W}^{\prime }\alpha_{1}^{\prime }$ are respectively the mean squares of discriminant scores when including the gene to be tested or not since, e.g. $\alpha_{1}^{T}M_{B}\alpha_{1}$ and $\alpha_{1}^{T}M_{W}\alpha_{1}$ are respectively the numerator and denominator in $F_{1}=\frac{\alpha_{1}^{T}S_{B}\alpha_{1}/(K-1)}{\alpha_{1}^{T}S_{W}\alpha_{1}/(N-K)}$. However, the exact same large sample test may not be appropriate for gene statistical redundancy testing here. *λ*_1_ and *λ′*_1_ are obtained when including a gene or not and thus the corresponding *F* ratios are not really independent. For this reason, we use a bootstrap test by directly estimating the null reference distribution of λ_1_/λ*′*_1_ with bootstrap re-sampling.

### Bootstrap re-sampling test for statistical redundancy

Assume there is a set of *m* genes, *C*, which can be described by a *m* × *N* data matrix *X_C_*. For each gene in *C*, a statistic λ_1_/λ*′*_1_ is calculated to evaluate its contribution to the discriminability of *C*. Under the null hypothesis that a gene is statistically redundant, the discriminability of the discriminant scores when applying the first discriminant function α_1_ to the data should not be significantly different whether including this gene in *C* or not. This means the discriminability of the discriminant scores should not be significantly different whether adding a statistically redundant gene or any one gene which has no individual discriminability to *C*. For gene *i*, under the null hypothesis *H*_0*i*_, we use the following algorithm to calculate the expected *λ*_1_/*λ′*_1_ and perform the statistical redundancy testing:

1. For each gene *i* in *C*, calculate the observed test statistic *λ*_1_/*λ′*_1_.
2. Generate *B* independent bootstrap sets *C^b^* where *b =* 1, ..., *B.* For gene *i* with real expression data (*x_i_*_1_, *x_i_*_2_, ..., *x_iN_*), re-sampling is done by groups with replacement to generate ( $x_{i1}^{b},\;\;x_{i2}^{b},\;\;\cdots ,\;\;x_{iN}^{b}$). The data of each gene is independently re-sampled to generate $X_{c}^{b}$ for *C^b^*. Repeat re-sampling until generate *B* independent sets $X_{c}^{1},\cdots X_{c}^{B}$.
3. For each gene *i* in *C^b^*, replace ( $x_{i1}^{b},\;\;x_{i2}^{b},\;\;\cdots ,x_{iN}^{b}$) with ( $x_{i1}^{*},x_{i2}^{*},\cdots ,x_{iN}^{*}$) where the latter is generated by re-sampling (*x_i_*_1_, *x_i_*_2_, ..., *x_iN_*) with replacement across the groups. In this way, data matrix $X_{c}^{b}$ is changed to ${\left(X_{c}^{b}\right)}_{i}$ and the latter is used to calculate $\lambda_{c}^{b}/\lambda_{c}^{b\prime }$ for this gene *i*.
  For gene *i*, calculate its bootstrap *p*-value as follows and set significant level as α to reject *H_i_* if *p̂_i_*≤α:
  $${\hat{p}}_{i}=\frac{1}{B}\sum_{b=1}^{B}I\;\left(\frac{\lambda_{1}}{\lambda_{1}^{\prime }}<\frac{\lambda_{1}^{b}}{\lambda_{1}^{b\prime }}\right)$$
4. Repeat steps 1~3 until all the genes in *C* are tested.
5. Apply a proper multiple test adjustment to control type I error.

### Gene selection methods

When the goal is selecting informative genes, the commonly used significant level like 0.05 may be too stringent and we might select very few genes with multiple test adjustment. This may be due to lack of samples or measurement errors in the samples. Instead, we can simply pick up genes with larger eigenvalue-ratios and sequentially remove statistically redundant or less important genes. In this section, we propose two gene selection methods using the eigenvalue-ratio statistic. One is to used when the number of genes is less than *N – K –* 2 and the other is to be used when the number of genes is larger than or equal to *N – K –* 2.

#### Algorithm 1

A forward selection method using statistical redundancy test (for *P < N – K –* 2):

1. Initialize *S* = {} as the set of selected genes and *C* = { *g*_(1)_, *g*_(2)_, …, *g*_(_*_M_*_)_} as the set of candidate genes, where *M = P*. Genes in *C* are sorted in the descending order using their absolute *t*-test values.
2. Pool genes in *S* and *C* together and compute eigenvalue-ratios of genes in *C*, ( *e*_(1)_, *e*_(2)_, …, *e*_(M)_). Compute the correlations of genes in *C* between *g*_(_*_j_*_)_s (*j* > 1) and *g*_(1)_(*c*_(2)_, *c*_(3)_, …, *c*_(_*_M_*_)_). Let *C*_1_ = {*g*_(_*_j_*_)_ | *g*_(_*_j_*_)_ ∈ *C*, *e*_(_*_j_*_)_ < *e*_(1)_ and *c*_(_*_j_*_)_ > *c_thresh_*}.
3. Select gene *S* = *S* + {*g*_(1)_} and remove gene *C* = *C – C*_1_.
4. Repeat step 2 to 3 until all genes have been either selected or removed, i.e. *C* = {}.
5. Return *S*.

In Algorithm 1, the absolute *t*-statistic is used to measure the individual discriminability of genes and genes are ordered in descending according to this measure. From the candidate gene set *C*, we first select the gene with the highest discriminability. Then the remaining candidate genes are tested against this selected gene using the eigenvalueratio statistic. Genes that have similar expression profile but show to be statistically redundant are expected to have lower eigenvalueratios than that of selected gene and therefore can be removed. This process continues until all genes are either selected or removed.

The correlation threshold *c_thresh_* is a non-negative value chosen from (0, 1) and to be used to make sure that only these genes that have similar expression levels to the selected gene will be tested and possibly removed. This is useful since, compared with the current selected gene, a gene selected earlier in the candidate set might have a very small eigenvalue-ratio if its statistically redundant genes exist in the set.

The value of *c_thresh_* controls how much statistical redundancy to be removed and therefore affects the size of *S*. A larger value of *c_thresh_* usually results in less genes being removed every time and therefore more genes being selected. A smaller value of usually results in more genes being removed every time and therefore less genes being selected.

Since *S_w_* is singular when *P > N* and FLDA suffers from numerical instability when *P ≈ N – K*, we use the following gene selection method by introducing hierarchical clustering to group genes with similar expression levels into smaller clusters and selecting genes within small clusters with Algorithm 1.

#### Algorithm 2

A forward selection method using clustering and statistical redundancy test (for *P ≥ N – K –* 2):

1. With DIANA, divisively cluster genes into a hierarchical tree using (1-Pearson correlation) as the distance measure. Any cluster in the hierarchy with a size larger than or equal to *N – K –* 2 should be divided into two sub-clusters. Initialize the correlation threshold *c_thresh_*.
2. Sort the sequence of nested clusters in the cluster hierarchy in the ascending order according to their sizes. Denote these clusters as *c_i_*, *i* = 1, 2, …, *I*.
3. From *i* = 1 to *I*, check the size of *c_i_*, i.e. the number of genes in *c_i_*. If the number of genes in the cluster is larger than or equal to *N – K –* 2, select genes in its sub-clusters using Algorithm 1 with *c_thresh_*. After gene selection of its sub-clusters, if the number of genes in the cluster is still larger than or equal to *N – K –* 2, quit the Algorithm and use a smaller value of *c_thresh_*.
4. Return genes in cluster *c_I_*.

The idea of Algorithm 2 is to group a large number of genes into small clusters with a size less than *N – K –* 2 so that Algorithm 1 can be used to remove the statistical redundancy. Since we sort clusters in an increasing order by the cluster size, a larger cluster always appears after its smaller sub-clusters. For a cluster with size larger than or equal to *N – K –* 2, we apply Algorithm 1 to its sub-clusters so that statistically redundant genes will be removed in the smaller sub-clusters. Then this cluster will have smaller size and can be processed later. At the end of processing, the last cluster, which originally contains all the genes, will now contain a compact gene set. We can also understand Algorithm 2 in a recursive way. If a cluster has a large number of genes (i.e. the number of genes is greater than *N – K –* 2) and cannot be processed by the Algorithm 1, we divide it into two smaller sub-clusters. If any of two sub-clusters still has too many genes, we split the sub-cluster again until a sub-cluster can be processed by the Algorithm 1. We recommend in Algorithm 2 that the gene selection starts when the number of genes is greater than *N – K –* 2. However, there is no doubt that we can specify a number which is less than *N – K –* 2.

When using Algorithm 2, it is likely that the total number of genes selected is still larger than or equal to *N – K –* 2 at the end of the whole gene selection process. In this case, we can build a classifier with generalized partial least squares method (Ding and Gentleman, 2005). Otherwise, we can use a smaller *c_thresh_* to select a smaller number of genes to build a classifier with logistic regression. To choose an appropriate *c_thresh_*, we can try a series of values in (0, 1), say (0.005, 0.01, 0.105, 0.02, …) and perform gene selection with the proposed methods. For each *c_thresh_*, we compute the leave-one-out cross-validation (LOO-CV) prediction error of the classifier built with the selected genes. Then the chosen *c_thresh_* is the one which gives the lowest LOO-CV prediction error.

## Simulations

### Simulation 1

Simulated 20 × 200 data matrix contains the expression data of 20 genes for two types of cancer where the first 100 samples are of type I and the other 100 samples are of type II. These genes are highly informative for separating the two cancer types. Some of the genes have very similar expression levels and the expression data are strongly correlated. When these correlated genes are used to build a classifier, some genes may subsume the effects of the others thus there will be redundancy problem. We use function “mvrnorm ()” in R package “MASS” to generate the data from a multivariate normal distribution. The data for gene 1 to gene 20 are generated by 2 groups as following: (1) group 1 includes gene 1~10 with each gene in type I distributed as *N*(0, 1), and the genes in type II distributed as *N*(μ_12_, 1) where μ_12_ = (0.3, 0.4, 0.5, 0.6, 0.7, 0.8, 0.9, 1.0, 1.1, 1.2) for gene 1~10; (2) group 2 includes gene 11~20 with each gene in type I distributed as *N*(0,1), and the genes in type II distributed as *N*(μ_22_, 1) where μ_22_ = (1.3, 1.4, 1.5, 1.6, 1.7, 1.8, 1.9, 2.0, 2.1, 2.2) for gene 11~20. The data are generated by sample types. For each sample type, the 2 groups are independent of each other and the pair-wise correlation between gene *i* and *j* is set to be corr(*i, j*) = 0.9^|i–j|^ within each group. Thus the closer the two genes in each group, the higher the correlation and the more similar their expression levels.

Statistical redundancy testing is performed by bootstrap re-sampling with *B* = 100 replications. Table 1 show the statistical redundancy test and *t*-test results for the simulated data. According to Table 1, gene 10 and gene 20 respectively have the greatest negative *t*-statistics in group 1 and group 2 thus both genes are individually most informative one in each group. The two genes have much larger eigenvalue-ratios than the other genes and are the only two genes to be tested as statistically non-redundant ones (*p*-value = 0 for gene 10; *p*-value = 0 for gene 20) at a significance level of 0.05 although the t-tests indicate that all of the genes in group 1 and 2 are individually highly informative. All the genes in group 2 are more informative than those in group 1 according to *t*-test, however, gene 10 in group 1 is still tested as non-redundant. The reason is that the two groups are independent of each other and contain very different information about the difference between the two cancer types, and thus the effect of gene 10 in group 1 can not be replaced by those in group 2 when we build classifiers with both groups of genes. Within each group, the pair-wise correlation is corr(*i, j*) = 0.9^|i–j|^ thus not only each pair of adjacent genes but also those genes not far away from each other have very similar expression levels and contain very similar information about the difference between the two cancer types, and even the two furthest genes (i.e. gene 1 and gene 10, gene 11 and gene 20) in each group have a correlation as high as 0.387. On the other hand, some of the correlated genes are individually less informative than the others. When correlated genes are used together in a classifier, the contribution of the individually less informative one to the joint discriminability may be negligible comparing to the contribution of the other individually more informative one. For these reasons, gene 1~9 and 11~19 should be tested as redundant.

Since we are testing multiple genes simultaneously, it is very critical to adjust for multiplicity. With BH false detection rate (FDR) control method (Benjamini and Hochberg, 1995), the statistical redundancy tests show gene 10 and gene 20 (adjusted *p*-value = 0) are non-redundant at an FDR level of 0.05.

### Simulation 2

Simulation 2 is about gene selection according to eigenvalue-ratio statistics using Algorithm 1. In this simulation, we still uses the data created in Simulation 1. Table 2.2 shows the sequence of the genes to be filtered with Algorithm 1 and the selected one at each gene removing step. Different correlation thresholds are tried for this simulation. When the correlation threshold is chosen as 0.4, gene 10 and 20 are selected as non-redundant ones and the logistic model with the two genes gives the lowest LOOCV. Table 2 shows the genes to be selected and the genes to be removed at each selection step. Considering the correlation matrix for simulation, it is obvious from Table 2.2 that 9 genes (19, 18, 17, 16, 15, 14, 13, 12 and 11) in group 2 are filtered as statistically redundant or less important ones when compared with gene 20 at step 1, and other 9 genes (9, 8, 7, 6, 5, 4, 3, 2 and 1) in group 1 are removed as statistically redundant or less important ones when compared with gene 10 at step 2. So both the statistical redundancy testing (with multiplicity adjustment) and the gene selection Algorithm 1 select the same genes for this simulated data. Such agreement between the two methods shows that Algorithm 1 is effective not only in removing statistically redundant genes but also finding the most important ones.

We also compare the performance of our gene selection method with Yu and Liu’s (2004) FCBF algorithm on the simulated data. Since the original method was proposed for covariates with discrete values and the method can not apply directly to gene expression data with continuous values, we discretize the data before using this method. With FCBF algorithm, gene 10 and 20 are selected and shown as statistically non-redundant ones thus the results agree with that obtained by Algorithm 1.

### Simulation 3

Simulation 3 is about gene selection with Algorithm 1 under different high pair-wise correlations. This simulation has a similar setup to Simulation 1 except that pair-wise correlations changes from high to low. The genes successively selected under different pair-wise correlations are shown in Table 3. Under each particular pair-wise correlation, the subset of genes selected with Algorithm 1 gives a logistic model with the lowest LOOCV misclassification error among all the models built with different correlation thresholds. Generally, more genes are selected with Algorithm 1 under lower pair-wise correlation. The reason is that even two non-adjacent genes may have very similar expression levels under a high pair-wise correlation, and the less informative one may result to be redundant when the other more informative one exists in the same classifier. In contrast, even the two adjacent genes do not have very similar expression levels under a low pair-wise correlation. For example, the correlation between gene 6 and the gene 10 is still as high as 0.656 when the pairwise correlation is 0.9, and much higher than that between gene 9 and gene 10 when the pair-wise correlation is 0.5.

Also shown in Table 3 are the genes selected with Yu and Liu’s (2004) FCBF algorithm. The two gene selection methods agree with each other when the pair-wise correlation is 0.9. As the pairwise correlation decreases, the two methods tend to select more and more different genes although they still select some common genes. At each pairwise correlation, Algorithm 1 selects the most informative gene from each group, i.e. gene 10 and gene 20. This method also selects one or more genes from both groups as the pair-wise correlation is or below 0.7. On the other hand, FCBF fails to select gene 10 when the pair-wise correlation is 0.8 and 0.5. FCBF only selects genes from group 2 when the pair-wise correlation is 0.5. This might be caused by the information loss during data discretization. Since the two groups are generated to be independent and genes in each group are very informative, we may expect the non-redundant genes to be representative of both groups, i.e. both groups have one or more genes to be selected. Thus Algorithm 1 generally outperforms FCBF for this simulation.

### Simulation 4

This simulation is about gene selection with Algorithm 2 when the number of genes is greater than the sample size. Simulated 400 × 100 data matrix contains the expression data of 400 genes for two types of cancer where the first 50 samples are of type I and the other 50 samples are of type II. In type I cancer, each gene is distributed as *N*(0,1). These genes are highly informative for separating the two cancer types. In type II cancer, genes are distributed as *N*(μ_2_, 1) where μ_2_[1:200] = (−1.996, −1.988, −1.980, …, 0.404) and μ_2_[201:400] = (0.404, 0.412, 0.420, …, 1.996). Data are generated by sample types and by blocks of genes. There are a total of 20 blocks and each block contains 20 genes with the first block contains gene 1~20, the second block contains gene 21~40 and so on. In each block of genes, we assume there are two independent groups of genes with 10 genes in each group and the pair-wise correlation between gene *i* and *j* is set to be corr(*i, j*) = 0.7^|i–j|^ within each group. For example, in block 1, gene 1~10 are in group 1 and gene 11~20 are in group 2, and, genes in group 1 and group 2 are independent.

Since genes are correlated in each group and the number of genes is much greater than the sample size, we use Algorithm 2 to reduce redundancy and select a compact gene set. Gene selection starts when the cluster size is greater than or equal to 40. When the correlation threshold is chosen as 0.4, the LOOCV prediction error of the logistic model is 0.01. Algorithm 2 selects 72 genes: 1, 10, 22, 52, 75, 81, 87, 118, 122, 126, 130, 131, 136, 141, 145, 147, 151, 152, 158, 161, 162, 168, 171, 175, 178, 180, 181, 184, 187, 190, 191, 194, 197, 200, 203, 204, 207, 210, 211, 212, 215, 217, 220, 221, 223, 226, 230, 231, 234, 238, 239, 244, 250, 255, 260, 269, 273, 284, 295, 300, 307, 313, 330, 351, 370, 400. When checking the correlations between the selected genes, we find out the following pairs of genes are pairs of adjacent genes in same groups in the whole correlation structure: 151 and 152, 161 and 162, 203 and 204, 211 and 212. Since the correlation between the pairs of adjacent genes in same groups is 0.7, there is still some redundancy in the selected 72-gene list. However, if we take the whole correlation structure into consideration, we can still observe Algorithm 2 removes most of redundancy for this simulated data.

## Gene Selection for Microarray Datasets

### Gene selection for leukemia study

The leukemia dataset used was published by Golub et al. (1999) and is available from http://www.broad.mit.edu/cgi-bin/cancer/datasets.cgi. The original research aimed to identify the most informative genes for the purpose of disease modeling and more accurate classification of acute lymphoblastic leukemia (ALL) and acute myeloid leukemia (AML). The training data set has 7129 genes, 27 cases of ALL and 11 cases of AML. The test data set has 20 cases of ALL and 14 cases of AML. The entire data set, i.e. the data of all the 72 samples, is used as training data to select genes in this paper. Pre-filtering according to *t*-test left 1110 genes that were significantly differentially expressed (*p*-value ≤ 0.01) for further gene selection.

Since there are 1110 genes but only 72 samples, we can not use Algorithm 1 directly but rather use Algorithm 2 for gene selection. Table 5 in Supplement materials show the 13 genes selected with Algorithm 2 with *c_thresh_*. = 0.2. HOXA9 is a genetic marker associated with myeloid cell lineage (Casas et al. 2003). MPO is one of the mostly important cytochemical stainings in acute leukemia. Clinically it has been used together with TdT to identify ALL and is an excellent “marker” gene for AML and ALL (Cortes and Kantarjian, 1997). Cell division hCDCrel-1 is a partner control related gene of MLL in some protein leukemias (Osaka, 1999). E2A is a known oncogene implicated in childhood B-ALL. NM23A is an oncogene and its higher level expression was correlated with poor prognosis for AML patients (Okabe-Kado et al. 1998).

Among the 13 genes, five genes are highly informative ones in Golub et al. (1999): M31523 (highly expressed in ALL); and, X95735, M27891, M55150 and U82759 (highly expressed in AML). Table 4 shows how Algorithm 2 dealt with Golub’s 50 genes. Each row shows a gene selection step where the selected gene in the left column has larger absolute *t*-statistic in the current gene set and the genes in the right column are those with smaller *t*-statistic and to be filtered as statistically redundant or less important ones. Ten of the selected genes in the left column and all of those in the right column are in Golub’s 50-gene list. Five genes in Golub’s list are selected at earlier steps but filtered as statistically redundant or less important ones at later steps: Z15115, X63469, Y00787, M91432 and M31211. Golub’s 50 genes are highly expressed either in ALL or AML. However, when these genes are evaluated together within the same set, some genes’ contribution is negligible when a more informative one exists. Some filtered genes are found to be strongly correlated with the selected genes so these are removed as statistically redundant ones. Some are not strongly correlated so removed as less important ones. Since genes involved in same biological pathways relevant with the disease may have similar expression profiles, it may be very interesting to find out the biological relationship between these filtered genes and those selected ones however this topic is far beyond the scope of this paper which focuses on statistical redundancy problem.

Among the 13 genes in Table 3, four are selected with FCBF method: X95735, M27891, M19507 and M31523; three are selected by Guyon et al. (2002) with SVM RFE and among their four top ranked genes: X95735, U82759 and U59632. To evaluate the joint discriminability of these 13 genes selected with Algorithm 2, we build a logistic model which yields zero LOOCV error. The logistic model built with the 13 genes selected with FCBF method yields one LOOCV error. Guyon et al. (2002) report zero LOOCV error for the genes that they found with SVM RFE.

### Gene selection for Colon cancer study

The colon cancer dataset was originally analyzed by Alon et al. (1999). This dataset contains expression levels of 2000 genes with highest minimal intensity across 40 tumor and 22 normal colon tissues. The data is available from R package “dprep”. Pre-filtering according to *t*-test left 851 genes that were significantly differentially expressed (*p*-value ≤ 0.1) for further gene selection.

Algorithm 2 selected 48 genes with *c_thresh_*. = 0.605. Logistic regression model based on this set yields 5 LOO-CV errors. Further selection from these 48 genes with Algorithm 1 gets a subset of 46 genes which can yield zero LOO-CV error. Table 6 in Supplement materials lists the information of the 46-gene subset. The human desmin gene was down-regulated in colon cancer tissues and also showed significantly reduced expression in melanoma cell line (Gutgemann et al. 2001). S–100P is also expressed in breast, prostate, and lung cancers. In colon cancer cell lines, its expression level was found to be correlated with resistance to chemotherapy (Bertram et al. 1998). Shailubhai et al.’s (2000) study demonstrated uroguanylin induced apoptosis in human colon carcinoma cells *in vitro*, and oral uroguanylin inhibits the formation of polyps in the *Min*/1 mouse animal model of colorectal cancer *in vivo*. IL-8 is responsible for tumor progression and liver metastasis of colorectal cancer (CRC), and activation of plasminogen activator system induced by IL-8 and VEGF may play important role in progression of CRC (Terada, et al. 2005). Fan et al.’s (2001) study found the down-regulation of matrix Gla protein mRNA generally occurs in colorectal adenocarcinomas. Bernini et al. (2000) reported that the MUC2 mucin gene was highly expressed in the colon and associated colorectal tumors and might be a candidate marker for colorectal cancer micro-metastases.

Among the 46 genes in Table 5, four are selected with FCBF method: M63391, M26383, R84411 and T47377; two are selected by Guyon et al. (2002) with SVM RFE and among their 7 top ranked genes: H08393 and H64807. To evaluate the joint discriminability of these 46 genes selected with Algorithm 2, we build a logistic model which yields zero LOOCV error. The logistic model built with the 7 genes selected with FCBF method yields 8 LOOCV error. Guyon et al. (2002) reported zero LOOCV error for the 7 genes that they found with SVM RFE.

## Discussion

In this paper, we defined a novel eigenvalue-ratio test statistic to quantitatively evaluate statistical redundancy and provide two gene selection methods using the test statistic. The eigenvalue-ratio test statistic is the ratio of the eigenvalue of including a gene to the eigenvalue of excluding the gene. It can be used to detect not only genes of low discriminability, but also those highly statistically redundant ones. We present a bootstrap re-sampling hypothesis testing based on the eigenvalue-ratio test statistic. We also develop a gene selection method using the eigenvalue-ratio test statistic, which sequentially selects genes and at the same time removes statistically redundant genes corresponding to the selected ones. In case of a large body of genes, our gene selection method can be combined with hierarchical divisive clustering to group genes into small clusters and then remove statistically redundant ones within small clusters. Since each cluster’s size is less than *N* when removing its redundant genes, the computational complexity of our gene selection method is *O*(*PN*^3^) when *P>>N*. In other words, our gene selection method has a complexity linear to *P* and therefore is a scalable approach. The simulation studies illustrate the problem of statistical redundancy and validate the proposed test statistic and gene selection methods. The high prediction accuracy and biological relevance of the selected genes for the two real data examples demonstrate the effectiveness of the proposed gene selection methods.

FLDA in the eigenvalue-ratio test statistic assumes linearity, but it can be extended to nonlinear manifolds using a nonlinear mapping such as kernels. In this paper, for simplicity we limit our test statistics and gene selection methods to two-class cancer classification problems. Future work might be further investigations to include multiple-class classification cases.

## Supplementary Materials

DIANA (Divisive Analysis) clustering Algorithm DIANA Algorithm is fully described by Kaufman and Rousseeuw (1990) and has been implemented in many statistical programming languages such as R and Splus. This Algorithm constructs a divisive hierarchy of clustering. That is, DIANA starts with one large cluster which contains the entire data set then progressively splits this initial cluster into smaller and smaller subsets until each cluster contains only a single observation.
At each stage of clustering, the cluster with the largest diameter will be selected where the diameter of a cluster is defined as the largest distances between any two of the observations in this cluster. For the selected cluster, DIANA Algorithm first looks for its most disparate observation (i.e. which has the largest average distance to the other observations of the selected cluster) then reassigns observations that are closer to this most disparate observation than to the original cluster and the average distances of the two clusters are recalculated. Suppose the selected cluster *C* has *n_c_* observations *x_i_* (*i*=1, …, *n*). At the *k*th iteration of reassigning observations, for observation *i*, the distance between *i* and the rest of the cluster *C* is defined as $\Delta_{k,x_{i}}=\frac{1}{n_{c}-1}\sum_{j\neq i,j\in C}$ *d*(*x*_i’_, and, if identifying the observation $x_{k}^{*}$ with the largest Δ*_k,i_* for the *k*th iteration, all such observations make a set C*. The iteration continues until the observations left in the original cluster are more similar to that cluster than C*. That is, the iteration stops when $\Delta_{k,x_{k}^{*}}<0$ where $\Delta_{k,x_{k}}=\frac{1}{n_{c}-k-1}\sum_{j\notin C^{*}}d(x_{k},x_{j})-\frac{1}{k-1}\sum_{l=1}^{k-1}d(x_{l}^{*},x_{j})$. Then cluster *C* is divided into two smaller clusters, one of which has the observations $x_{l}^{*}$ (*l* = 1, …, *k –* 1) in cluster *C* and the other has the remaining observations in cluster *C*.
The result obtained with DIANA is a matrix of cluster membership per Algorithm iteration. Given the number of clusters, this matrix can be sorted and the cluster membership of the observations can be found.

FLDA Fisher Linear discriminant analysis (FLDA) finds a reduced set of new dimensions on which projected data has maximal discriminability among classes and has been used as a supervised dimension reduction technique. FLDA attempts to minimize the Bayes classification error by searching for an optimal linear combination α of the predictors which maximizes the product of the inverse of the within-class variance matrix and the between-class variance matrix. Such α’ s can be given by the largest eigenvalues *λ′s* of *S_W_*^−1^ *S_B_* by solving a generalized eigenvalue problem, i.e. $\lambda =\text{max}\frac{\alpha^{T}S_{B}\alpha }{\alpha^{T}S_{W}\alpha }$, where *S_B_* is between-class variance matrix and *S_W_* is within-class covariance matrix. For *k*-class classification, there are at most *K–*1 non-zero eigenvalues and *K–*1 corresponding orthogonal discriminant functions. When P > N, FLDA can not be used directly because of the singularity problem, i.e. *S_W_* is singular in this case.
In this paper, we focus on two-class classification for simplicity, i.e. *K* = 2. Thus there is only one non-zero λ and one corresponding α that can be denoted as λ_1_ and α_1_ respectively.

Selected genes for Leukemia data

**Table 5.** The 13 selected genes for Leukemia data.

**Access number**	**Description**
X95735	Zyxin
M27891	CST3 Cystatin C (amyloid angiopathy and cerebral hemorrhage)
M55150	FAH Fumarylacetoacetate
M19507	MPO Myeloperoxidase
U82759	GB DEF = Homeodomain protein HoxA9 mRNA
U59632	Cell division control related protein (hCDCrel-1) mRNA
X57398	NME1 Non-metastatic cells 1, protein (NM23A) expressed in isoform a
M31523	TCF3 Transcription factor 3 (E2A immunoglobulin enhancer binding factors E12/E47)
M11722	Terminal deoxynucleotidyl transferase mRNA (TdT)
U52682	IRF4 Interferon regulatory factor 4
HG651–HT4201	Adducin, Alpha Subunit, Alt. Splice 2
U51010	GB DEF = Nicotinamide N-methyltransferase gene, exon 1 and 5′ flanking region
M37457	Na+,K+ -ATPase catalytic subunit alpha-III isoform gene

Selected genes for Colon cancer data

**Table 6.** The 46 selected genes for Colon cancer data.

**Access number**	**Discription**
H08393	COLLAGEN ALPHA 2(XI) CHAIN (Homo sapiens)
M63391	Human desmin gene, complete cds
T47377	S-100P PROTEIN (HUMAN)
R84411	SMALL NUCLEAR RIBONUCLEOPROTEIN ASSOCIATED PROTEINS B AND B’ (HUMAN)
Z50753	H.sapiens mRNA for GCAP-II/uroguanylin precursor
H55916	PEPTIDYL-PROLYL CIS-TRANS ISOMERASE, MITOCHONDRIAL PRECURSOR (HUMAN)
T59878	PEPTIDYL-PROLYL CIS-TRANS ISOMERASE B PRECURSOR (HUMAN)
H17434	NUCLEOLIN (HUMAN)
M26383	Human monocyte-derived neutrophil-activating protein (MONAP) mRNA, complete cds
T60778	MATRIX GLA-PROTEIN PRECURSOR (Rattus norvegicus)
M82919	Human gamma amino butyric acid (GABAA) receptor beta-3 subunit mRNA, complete cds
R42244	ANTIGEN PEPTIDE TRANSPORTER 1 (HUMAN)
M80815	H.sapiens a-L-fucosidase gene, exon 7 and 8, and complete cds.
R62549	PUTATIVE SERINE/THREONINE-PROTEIN KINASE B0464.5 IN CHROMOSOME III (Caenorhabditis elegans)
T72863	FERRITIN LIGHT CHAIN (HUMAN)
T41204	P14780 92 KD TYPE V COLLAGENASE PRECURSOR
X68688	H.sapiens ZNF33B gene
T61661	PROFILIN I (HUMAN)
R52081	TRANSCRIPTIONAL ACTIVATOR GCN5 (Saccharomyces cerevisiae)
D26129	RIBONUCLEASE PANCREATIC PRECURSOR (HUMAN); contains element MER21 repetitive element
T47383	ALKALINE PHOSPHATASE, PLACENTAL TYPE 1 PRECURSOR (Homo sapiens)
R80427	C4-DICARBOXYLATE TRANSPORT SENSOR PROTEIN DCTB (Rhizobium leguminosarum)
M28128	Homo sapiens eosinophil cationic protein (ECP) mRNA, complete cds
R73660	GAMMA-INTERFERON-INDUCIBLE PROTEIN IP-30 PRECURSOR (HUMAN)
M96839	Human proteinase 3 gene, exon 5 and cds (3′ end)
R65697	ATP SYNTHASE A CHAIN (Trypanosoma brucei brucei)
H64807	PLACENTAL FOLATE TRANSPORTER (Homo sapiens)
K02268	Human enkephalin B (enkB) gene, exon 4 and 3′ flank and complete cds
H73908	METALLOTHIONEIN-IA (Bos taurus)
M28373	Homo sapiens amyloid protein A4 precursor mRNA, 3′ end of cds
M94132	Human mucin 2 (MUC2) mRNA sequence
M23419	INITIATION FACTOR 5A (HUMAN);contains element PTR5 repetitive element
J03210	Human collagenase type IV mRNA, 3′ end
T53396	60S ACIDIC RIBOSOMAL PROTEIN P1 (Polyorchis penicillatus)
T72175	IG KAPPA CHAIN PRECURSOR V-III REGION (HUMAN)
M29277	Human isolate JuSo MUC18 glycoprotein mRNA (3′ variant), complete cds
M85289	Human heparan sulfate proteoglycan (HSPG2) mRNA, complete cds.
R28373	HEMOGLOBIN BETA CHAIN (HUMAN)
T67406	COMPLEMENT C4 PRECURSOR (Homo sapiens)
T57882	MYOSIN HEAVY CHAIN, NONMUSCLE TYPE A (Homo sapiens)
H02465	GUANINE NUCLEOTIDE-BINDING PROTEIN G(I)/G(S)/G(O) GAMMA-7 SUBUNIT (Bos taurus)
M59807	NATURAL KILLER CELLS PROTEIN 4 PRECURSOR (HUMAN); contains element MSR1 repetitive element
R38636	UROKINASE PLASMINOGEN ACTIVATOR SURFACE RECEPTOR, GPI-AN CHORED (HUMAN)
T57780	IG LAMBDA CHAIN C REGIONS (HUMAN)
R33481	TRANSCRIPTION FACTOR ATF-A AND ATF-A-DELTA (Homo sapiens)
M31994	Human cytosolic aldehyde dehydrogenase (ALDH1) gene, exon 13

## Figures and Tables

**Table 1.** Gene *p*-values for statistical redundancy test and *t*-test.

**Gene**	***t*-statistic**	**Eigenvalue-ratio**	***p*-value for Statistical Redundancy test**	**Adjusted *p*-value with BH method**
1	−2.12132	1.002851	0.57	0.98
2	−2.82843	1.000007	0.98	0.98
3	−3.53553	1.000011	0.95	0.98
4	−4.24264	1.000016	0.98	0.98
5	−4.94975	1.000021	0.96	0.98
6	−5.65685	1.000028	0.96	0.98
7	−6.36396	1.000035	0.93	0.98
8	−7.07107	1.000044	0.92	0.98
9	−7.77817	1.000053	0.92	0.98
10	−8.48528	1.03608	0	0
11	−9.19239	1.001265	0.7	0.98
12	−9.89949	1.000086	0.95	0.98
13	−10.6066	1.000098	0.85	0.98
14	−11.3137	1.000112	0.9	0.98
15	−12.0208	1.000126	0.9	0.98
16	−12.7279	1.000141	0.88	0.98
17	−13.435	1.000158	0.86	0.98
18	−14.1421	1.000175	0.83	0.98
19	−14.8492	1.000192	0.85	0.98
20	−15.5563	1.082116	0	0

**Table 2.** Gene filtering process in Algorithm 1.

**Step**	**Genes to be selected**	**Genes to be filtered**
1	20	19, 18, 17, 16, 15, 14, 13,12,11
2	10	9, 8, 7, 6, 5, 4, 3, 2, 1

**Table 3.** Genes selected under different pair-wise correlations.

**corr*(i, j)***	**Algorithm**	**1FCBF**
0.9^|i-j|^	20 10	20 10
0.8^|i-j|^	20 10 4	20 16 11 9
0.7^|i-j|^	20 11 10 6 3	20 17 15 12 10
0.6^|i-j|^	20 11 10 7 4 2	20 18 16 14 10
0.5^|i-j|^	20 14 11 10 8 6 4 2	20 19 17 15 13 11

**Table 4.** Golub’s 50 genes that are selected and/or filtered.

**Genes to be selected**	**Genes to be filtered**				
Z15115	X15949				
X63469	U20998				
X90858	U46751	M57710	M80254		
Y00787	M28130	L08246	M69043		
M91432	X74262	U32944			
M27891	M63138	M83652	M19045		
X95735	M23197	M84526	M16038	X17042	M62762
M11722	M92287	U05259			
M55150	U50136				
M32304	M81695				
M31211	X59417	M91432	U22376	U26266	D38073
M31523	Z15115				
HG1612-HT1612	M29696	L47738	D26156		
X95735	X04085				
M19507	M96326				
M13792	M31303	U35451			
M31523	Y08612	M31211	U29175	X63469	Z69881
M27891	Y00787	Y12670			
M19507	X85116				
M31523	S50223	M13792			
